# M_1_ and M_3_ muscarinic receptors may play a role in the neurotoxicity of anhydroecgonine methyl ester, a cocaine pyrolysis product

**DOI:** 10.1038/srep17555

**Published:** 2015-12-02

**Authors:** Raphael Caio Tamborelli Garcia, Livia Mendonça Munhoz Dati, Larissa Helena Torres, Mariana Aguilera Alencar da Silva, Mariana Sayuri Berto Udo, Fernando Maurício Francis Abdalla, José Luiz da Costa, Renata Gorjão, Solange Castro Afeche, Mauricio Yonamine, Colleen M. Niswender, P. Jeffrey Conn, Rosana Camarini, Maria Regina Lopes Sandoval, Tania Marcourakis

**Affiliations:** 1 Department of Clinical and Toxicological Analysis, School of Pharmaceutical Sciences, University of São Paulo, Av. Prof. Lineu Prestes, 580, Bl. 13B, 05508-000, São Paulo/SP, Brazil; 2 Institute of Environmental, Chemical and Pharmaceutical Sciences, Federal University of São Paulo, Rua São Nicolau, 210, 1° andar, 09913-030, Diadema/SP, Brazil; 3 Laboratory of Pharmacology, Butantan Institute, Av. Vital Brasil, 1500, 05503-900, São Paulo/SP, Brazil; 4 Criminalistic Institute of São Paulo, Rua Moncorvo Filho, 410, 05507-060, São Paulo/SP, Brazil; 5 Institute of Physical Activity Sciences and Sports, Post-Graduate Program in Human Movement Sciences, Cruzeiro do Sul University, São Paulo, Brazil; 6 Department of Pharmacology, Vanderbilt University Medical Center, Nashville, TN 37212, USA; 7 Vanderbilt Center for Neuroscience Drug Discovery, Vanderbilt University Medical Center, 2201 West End Avenue, 1205 Light Hall, 37232-0697, Nashville/TN, USA; 8 Department of Pharmacology, Institute of Biomedical Sciences, University of São Paulo, Av. Prof. Lineu Prestes, 1524, Prédio 1, 05508-900, São Paulo/SP, Brazil

## Abstract

The smoke of crack cocaine contains cocaine and its pyrolysis product, anhydroecgonine methyl ester (AEME). AEME possesses greater neurotoxic potential than cocaine and an additive effect when they are combined. Since atropine prevented AEME-induced neurotoxicity, it has been suggested that its toxic effects may involve the muscarinic cholinergic receptors (mAChRs). Our aim is to understand the interaction between AEME and mAChRs and how it can lead to neuronal death. Using a rat primary hippocampal cell culture, AEME was shown to cause a concentration-dependent increase on both total [^3^H]inositol phosphate and intracellular calcium, and to induce DNA fragmentation after 24 hours of exposure, in line with the activation of caspase-3 previously shown. Additionally, we assessed AEME activity at rat mAChR subtypes 1–5 heterologously expressed in Chinese Hamster Ovary cells. l-[N-methyl-^3^H]scopolamine competition binding showed a preference of AEME for the M_2_ subtype; calcium mobilization tests revealed partial agonist effects at M_1_ and M_3_ and antagonist activity at the remaining subtypes. The selective M_1_ and M_3_ antagonists and the phospholipase C inhibitor, were able to prevent AEME-induced neurotoxicity, suggesting that the toxicity is due to the partial agonist effect at M_1_ and M_3_ mAChRs, leading to DNA fragmentation and neuronal death by apoptosis.

Cocaine, a tropane alkaloid found in *Erythroxylum coca* leaves, is a recreational drug used worldwide. Its abuse is a public health problem, affecting 17 million people (range 14–21 million) during 2012, which corresponds to a 0.4% rate of annual prevalence. Cocaine usage occurs with high incidence in North and South America (1.8 and 1.2% annual prevalence rates, respectively), Oceania (1.5%), and Western and Central Europe (1%)^1^. It blocks the uptake of serotonin, norepinephrine and dopamine in presynaptic nerve terminals, as well as voltage-specific sodium channels, responsible for the local anesthetic effect^2^. Cocaine may cause intense vasoconstriction, endothelial cell dysfunction, oxidative stress and platelet aggregation^3^,^4^,^5^, which are responsible for the main systemic adverse effects of its abuse, including stroke, myocardial infarction, arterial dissection, vascular thrombosis, rhabdomyolisis and renal complications^6^,^7^.

There are two distinct chemical forms of cocaine: hydrochloride (‘street’ cocaine, ‘coke’), a water-soluble powder which can be taken orally, intranasally or intravenously; and ‘freebase’ or ‘crack’ cocaine, which is cocaine without the hydrochloride moiety^8^,^9^. Crack cocaine is the smoked form of cocaine and has greater addictive potential than other routes of cocaine administration^10^. As crack cocaine has a low melting point (96–98 °C), the heating process quickly volatizes the cocaine, which is rapidly absorbed by the lungs and reaches the brain faster than any other route. Along with cocaine, anhydroecgonine methyl ester (AEME), a cocaine pyrolysis product, is also absorbed by the lungs^11^. Up to 80% of cocaine can be converted to AEME, depending on the temperature, the purity of the crack cocaine and the smoking devices^12^.

Little is known about AEME effects. To the best of our knowledge, the data available about this substance thus far includes only studies in the peripheral system, i.e., reductions in blood pressure and heart rate in rabbits with an increase in the respiratory rate^13^; a negative inotropic effect *in vitro* possibly mediated by muscarinic cholinergic receptors (mAChRs), since it was reversed by atropine, a nonspecific muscarinic receptor antagonist^14^; cardiovascular effects, e.g., hypotension and tachycardia in sheep, which are also antagonized by intravenous administration of atropine, again consistent with a muscarinic cholinergic effect^15^. However, the mechanisms of AEME in the central nervous system are poorly investigated and not well understood.

Our group first described the neurotoxicity of AEME and also the involvement of mAChRs in AEME-induced neuronal death in rat primary hippocampal cell cultures^16^. AEME seems to be a neurotoxic agent with greater neurotoxic potential than cocaine, showing an additive effect when combined. Caspase-3 activity in the hippocampal neurons was increased after 6 hours of exposure and seems to be one of the main mechanisms of AEME-induced neurotoxicity. Also, atropine prevented AEME-induced neurotoxicity, reinforcing that mAChRs are involved in AEME’s effects. In addition, binding experiments with hippocampi membrane preparations have confirmed the affinity of AEME for muscarinic cholinergic receptors^16^.

The mAChRs belong to the G-protein coupled receptors and include five distinct subtypes, denoted as M_1_-M_5_ mAChRs, which are widely distributed throughout the body. The odd-numbered mAChRs subtypes are coupled to G_q_/G_11_ proteins and induce the hydrolysis of phosphoinositide lipids by phospholipases, while M_2_ and M_4_ mAChRs subtypes, coupled to G_i_/G_0_ proteins, inhibit adenylyl cyclase activity. Thus, the activation of M_1_, M_3_ and M_5_ mAChRs subtypes produces inositol trisphosphate (IP_3_) and intracellular calcium, while M_2_ and M_4_ mAChRs subtypes cause a downstream decrease in cAMP levels^17^,^18^. It is important to emphasize that all five mAChRs are expressed in the hippocampus^19^,^20^ and they modulate hippocampal function through the inhibition of synaptic activity and/or the increase of neuronal excitability^21^.

To better understand the interaction between AEME and mAChRs, the present study investigated: 1) the effects of AEME on the production of total inositol phosphate and intracellular calcium release in rat primary hippocampal cell cultures, as well as DNA fragmentation as a result of caspase-3 activation, which was previously observed by our group^16^; 2) the affinity and the action of AEME at mAChRs using Chinese hamster ovary (CHO) cells expressing all five individual rat mAChRs subtypes; 3) the effects of M_1_ and M_3_ selective antagonists on AEME-induced neurotoxicity, as well as the inhibition of phospholipase C (PLC). The comprehension of the mechanisms underlying AEME-induced neurotoxicity could contribute to explain why crack cocaine smoke is more devastating than other routes of cocaine administration.

## Results

### Experiments with rat primary hippocampal cell culture

#### Effect of AEME on total [^3^H]inositol phosphates accumulation and on intracellular calcium release

**Figure 1:**
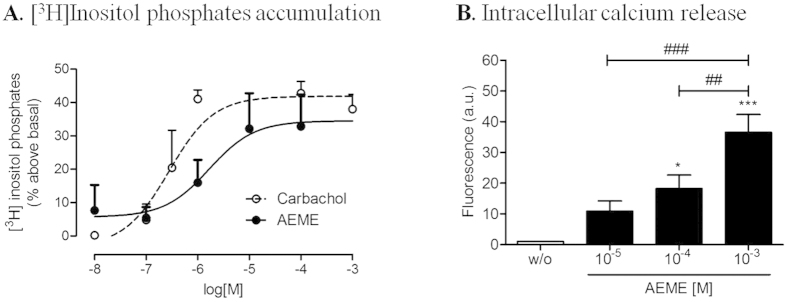
Effect of AEME on total [^3^H]inositol phosphates accumulation and on intracellular calcium release in hippocampal cell after culturing. (**A)** Concentration-effect curves of carbachol and AEME on total [^3^H]inositol phosphate accumulation in hippocampal cells. Maximum inositol phosphate accumulation was obtained with 10^−5^ M (10 μM) AEME and carbachol. Each point and vertical line represent the mean ± SEM of three to five experiments performed in duplicate. The basal level of the total [^3^H]inositol phosphate was 30626 ± 4250 dpm/10^6^ cells. (**B**) Intracellular calcium release after exposure to 10^−5^, 10^−4^ and 10^−3^ M AEME. w/o: without AEME. ^*^p < 0.05 and ^***^p < 0.001, compared with control group (w/o), ^##^*p* < 0.01 and ^###^*p* < 0.001 intergroup comparison (ANOVA and Newman-Keuls multiple comparison). Each bar and vertical line represent the mean ± SEM of ten independent experiments, each one performed in duplicate.

AEME and the cholinergic agonist carbachol (control) (10^−8^ to 10^−3^ M) caused a concentration-dependent increase of total [^3^H]inositol phosphate in the primary hippocampal cell culture. Maximum inositol phosphate accumulation was obtained with 10^−5^ M (10 μM) AEME and carbachol (Fig. 1A). The basal level of the total [^3^H]inositol phosphate was 30626 ± 4250 dpm/10^6^ cells.

There was a concentration-dependent increase in intracellular calcium release after AEME exposure (Fig. 1B), with the lowest effect observed with 10^−4^ M AEME and highest with 10^−3^ M AEME (F_3,36_ = 13.76; *p* < 0.001).

#### Effect of AEME on DNA fragmentation

**Figure 2:**
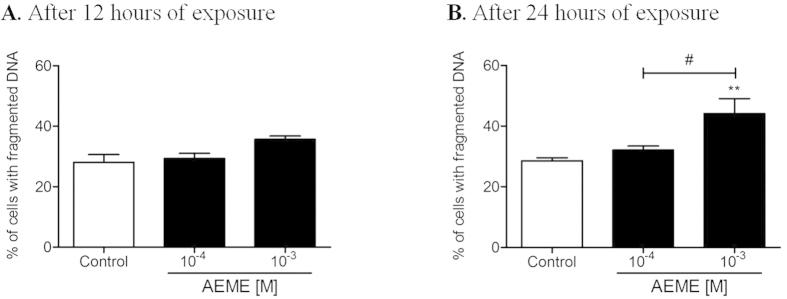
Percentage of hippocampal cells with fragmented DNA after exposure to AEME for: (**A**) 12 and (**B**) 24 hours. Each bar and vertical line represent the mean ± SEM of three to four independent experiments. ^**^p < 0.01, compared with control group, ^#^*p* < 0.05 intergroup comparison (ANOVA and Newman-Keuls multiple comparison).

Hippocampal cells exposed either to 10^−4^ or 10^−3^ M AEME for 12 hours (Fig. 2A) showed the same pattern of DNA fragmentation as the control group (F_2,6_ = 4.73; *p* = 0.059). However, after 24 hours of exposure, 10^−3 ^M AEME increased the number of cells with fragmented DNA (44.1%; F_2,8_ = 9.77; *p* = 0.007) compared to control (28.6%) and the lower AEME concentration (32.1%; Fig. 2B).

### Experiments with CHO cells

#### B_max_ and K_d_ determination for each mAChR subtype expressed in CHO cells

The saturation binding of [^3^H]NMS for each mAChR subtype expressed in CHO cells was specific and saturable and the specific binding fitted best a one-site model (see Supplementary Figure S1). An analysis of three experiments, each one performed in triplicate yielded dissociation constant (K_d_) and binding capacity (B_max_), summarized in Table 1.

#### Radioligand competition binding

**Figure 3:**
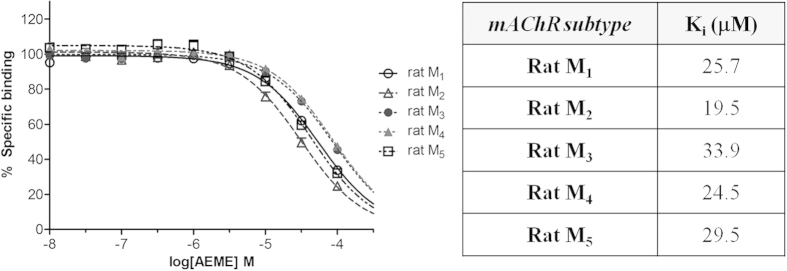
AEME competition binding curves obtained in CHO cells expressing individual subtypes of muscarinic receptors (data represent the mean of three independent experiments performed in triplicate). The K_i_ values for AEME (μM) were plotted alongside with the competition binding curves. The affinity for rat M_2_ was greater than rat M_4_ (*p* < 0.05), rat M_4_ was greater than rat M_3_ (p < 0.05), and rat M_1_ greater than rat M_3_ (p < 0.05). There was no difference among rat M_4_, rat M_1_ and rat M_5_. The averages of absolute values of controls are: 2274 ± 187, 888 ± 25, 3698 ± 303, 2831 ± 231, and 4059 ± 415 dpm/25 μg of protein for rat M_1_, rat M_2_, rat M_3_, rat M_4_ and rat M_5_, respectively. Data presented as mean ± SEM.

[^3^H]NMS displacement curves for AEME are shown in Fig. 3. The AEME K_i_ values (μM) were: 25.7 for rat M_1_, 19.5 for rat M_2_, 33.9 for rat M_3_, 24.5 for rat M_4_, and 29.5 for rat M_5_. The one-way ANOVA showed that the affinity for rat M_2_ was greater than rat M_4_; rat M_4_ greater than rat M_3_; and rat M_1_ greater than M_3_ (*p* < 0.05). Other than this observation, there was no difference among rat M_4_, rat M_1_ and rat M_5_, and no difference between rat M_5_ and rat M_3_. The averages of absolute values of controls are: 2274 ± 187, 888 ± 25, 3698 ± 303, 2831 ± 231, and 4059 ± 415 dpm/25 μg of protein for rat M_1_, rat M_2_, rat M_3_, rat M_4_ and rat M_5_, respectively.

#### AEME functional assays

**Figure 4:**
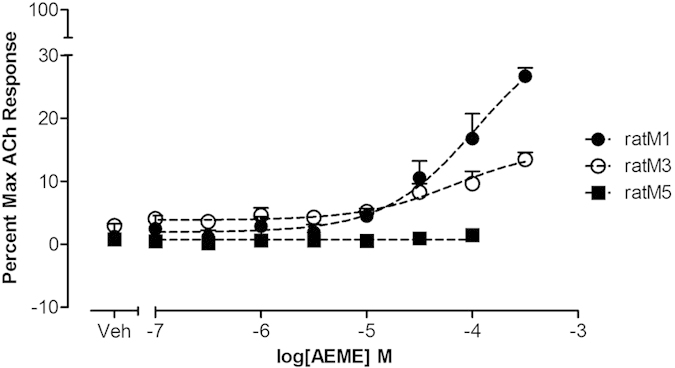
AEME concentration-response curve normalized to the maximum acetylcholine response. CHO cells expressing rat M_1_, rat M_3_ and rat M_5_ were studied (data represent the mean of three independent experiments, each one performed in triplicate). The AEME concentration effect was greater than 100 μM for both rat M_1_ and rat M_3_. The averages of absolute values of control are: 21950 ± 133, 23107 ± 77, and 20117 ± 1323 arbitrary units/4 × 10^4^ cells for rat M_1_, rat M_3_ and rat M_5_, respectively. Data presented as mean ± SEM.

**Figure 5:**
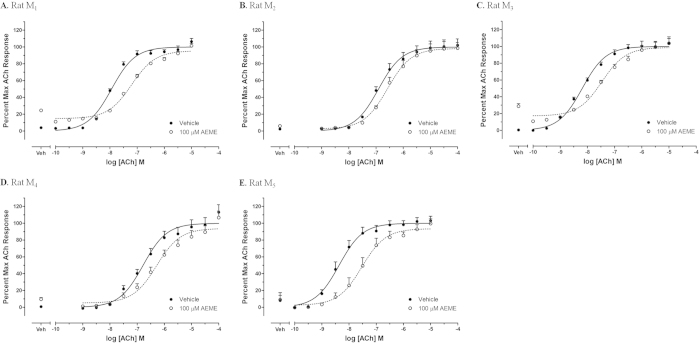
Calcium mobilization assay in CHO-K1 cells stably expressing all five subtypes of muscarinic receptors (data represent the mean of three independent experiments, each one performed in duplicate): rat M_1_ (**A**), rat M_2_ (**B**), rat M3 (**C**), rat M_4_ (D) and rat M_5_ (**E**). The averages of absolute values of control are: 20307 ± 149, 18233 ± 86, and 23438 ± 205 arbitrary units/4 × 10^4^ cells for rat M_1_, rat M_3_ and rat M_5_, respectively; 21937 ± 178 and 20218 ± 165 arbitrary units/6 × 10^4^ cells for rat M_2_ and rat M_4_, respectively. Data presented as mean ± SEM.

Calcium mobilization assays (Figs 4 and 5) were performed to determine AEME’s mechanism of action. AEME exhibited partial agonist-activity at the M_1_ and M_3_ mAChRs subtypes as observed by an increase in intracellular calcium fluorescence. The effective concentration that increased the response to 50% of maximum (EC_50_) was greater than 100 μM for both rat M_1_ and rat M_3_, reaching 38.3% (rat M_1_) and 27.2% (rat M_3_) of the maximum acetylcholine response (Fig. 4). There was no calcium increase at rat M_5_. The averages of absolute values of control are: 21950 ± 133, 23107 ± 77, and 20117 ± 1323 arbitrary units/4 × 10^4^ cells for rat M_1_, rat M_3_ and rat M_5_, respectively.

The effects of 100 μM AEME on acetylcholine concentration-response curves are shown in Fig. 5. Weak partial agonist activity at rat M_1_ and rat M_3_ was confirmed by the presence of an increase in calcium fluorescence at low concentrations of acetylcholine (Fig. 5A,C). AEME antagonized the effects of ACh at rat M_2_, rat M_4_, and rat M_5_ (Fig. 5B,D,E; EC_50_s are summarized in Table 2). In light of the ability of AEME to displace NMS, this suggested potential antagonist activity of AEME at rat M_5_. The averages of absolute values of controls are: 20307 ± 149, 18233 ± 86, and 23438 ± 205 arbitrary units/4 × 10^4^ cells for rat M_1_, rat M_3_ and rat M_5_, respectively; 21937 ± 178 and 20218 ± 165 arbitrary units/6 × 10^4^ cells for rat M_2_ and rat M_4_, respectively.

**Figure 6:**
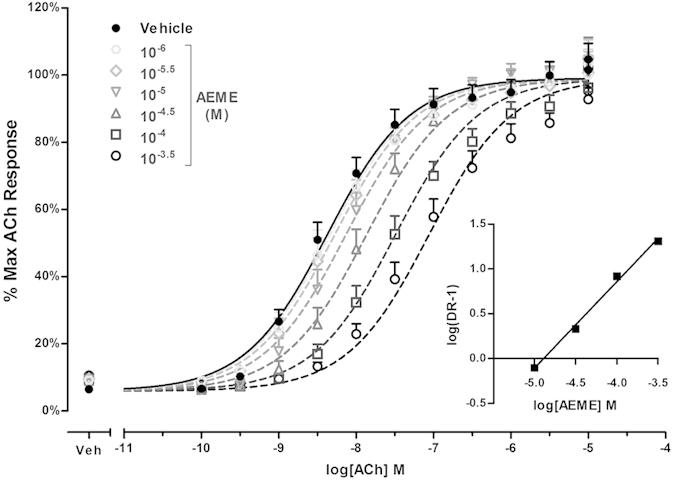
Acetylcholine concentration-response curves in the absence or presence of different concentrations of AEME (10^−6^ to 10^−3.5^ M) in a calcium mobilization assay using CHO-K1 cells stably expressing rat M_5_. Schild regression of the dose ratios (DR) derived from the AEME antagonism of acetylcholine was presented and the slope was 0.95 ± 0.07. The average of absolute values of control is 21255 ± 124 arbitrary units/4 × 10^4^ cells. Data presented as mean ± SEM. ACh, acetylcholine.

As the AEME antagonist effect appeared most robust at the rat M_5_ mAChR subtype, Schild analyses were performed to determine antagonist affinity (pA2). Using this technique, we estimated the pA2 and Schild slopes, which were 4.87 ± 0.10 and 0.95 ± 0.07, respectively (Fig. 6). These results suggest that AEME is a competitive orthosteric antagonist at rat M_5_. The average of absolute values of control is 21255 ± 124 arbitrary units/4 × 10^4^ cells.

### Effect of M_1_ and M_3_ selective mAChR antagonists and the PLC inhibitor on AEME-induced neurotoxicity

**Figure 7:**
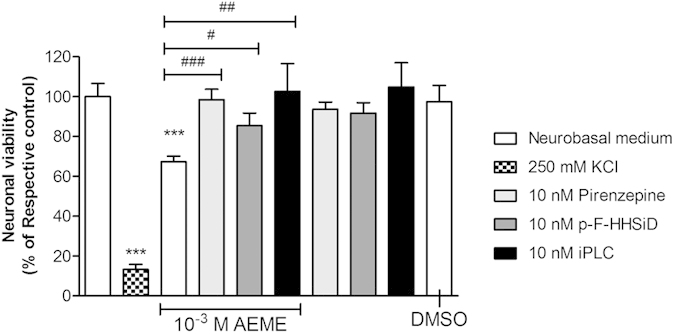
Effect of M_1_ selective antagonist pirenzepine (10 nM), M_3_ selective antagonist p-fluoro-hexahydrosila-difenidol (p-F-HHSiD, 10 nM) and PLC inhibitor (U73122, 10 nM) after 24 hours of exposure to 10^−3 ^M AEME (n = 4). Potassium chloride (250 mM) was used as a positive control of neuronal death. ^***^p < 0.001, compared with neurobasal medium (control group), ^#^*p* < 0.05, ^##^*p* < 0.01, ^###^*p* < 0.001compared with 10^−3^ M AEME (ANOVA and Newman-Keuls multiple comparison).

MTT viability assay (Fig. 7) was performed to determine the involvement of either M_1_ or M_3_ mAChRs, as well as the PLC inhibitor (U73122) on AEME-induced neurotoxicity. After 24 hours of exposure, 10^−3^ M AEME presented a significant decrease in neuronal viability, presenting 67.2% of viable cells (F_9,220_ = 20.87; *p* < 0.001). The incubation of AEME in the presence of the M_1_ selective antagonist, pirenzepine, prevented its neurotoxicity (98.4% of viable cells; *p* < 0.001 compared with AEME), as well as with the M_3_ selective antagonist, p-fluorohexahydro-sila-difenidol hydrochloride (p-F-HHSiD) (85.4% of viable cells; p < 0.05 compared with AEME) and iPLC (U73122) (102.6% of viable cells; *p* < 0.01 compared with AEME).

## Discussion

This manuscript comprises the first description of the mechanisms of AEME at mAChRs. In saturation binding experiments with [^3^H]NMS, we observed monophasic curves, allowing us to determine B_max_ and K_D_ values for individual mAChR subtypes. To characterize the effect of AEME at these receptors, different concentrations of AEME were examined for their ability to compete with [^3^H]NMS for its binding site. The binding curves in competition experiments with AEME were indicative of a single binding site for [^3^H]NMS. The range of pK_i_ values obtained for AEME in CHO cells from rat M_1_ to rat M_5_ was comparable with pK_i_ values for AEME from our previous studies with [^3^H]quinuclidinyl benzilate ([^3^H]QNB; 4.15  ± 0.15) in rat hippocampus^16^, indicating that mAChRs may be a target of AEME. Although the pKi values were very similar, we observed a slight preference for the M_2_ mAChR.

In the hippocampus of male rats, immunoprecipitation studies indicated a predominance of the M_1_ subtype (55%) and low expression of M_2_ (17%), M_3_ (10%) and M_4_ (15%)^22^. Their order of abundance has been ranked as M_1_ ≫ M_2_>M_3_ = M_4_>M_5_^19^,^20^. In hippocampus of female rats in proestrus, immunoprecipitation studies also confirmed that, although hippocampus expresses all mAChR subtypes, the population of M_1_ receptors is predominant. In addition, the amount of M_2_ in the hippocampus is higher than the amount of M_3_, M_4_ and M_5_ mAChRs^20^. Considering that the expression of M_1_ receptor is higher than the others mAChR subtypes in rat hippocampus^19^,^20^,^22^, in the present study we showed that this substance is a partial agonist at M_1_ and M_3_ mAChRs subtypes through the increase in the intracellular calcium, even in the presence of low concentrations of acetylcholine. Also, 100 μM AEME slightly shifted the acetylcholine concentration-response curves to the right for the M_2_ and M_4_ mAChR subtypes, characterizing a weak antagonist effect. The same antagonist effect was also observed for M_5_ mAChR; however, the effect of AEME seems to be relatively higher for mAChRs with lower EC_50_ for acetylcholine. It is important to note that AEME increased, in a concentration-dependent manner, total [^3^H]inositol phosphate, indicating that AEME may act as an agonist at the M_1_ and/or M_3_ mAChR subtypes.

The intracellular cascade involved in the activation of the M_1_ mAChRs increases the intracellular calcium^23^, which in turn might be involved in the activation of the caspase signaling, leading to neuronal death. In fact, Shih *et al.*^24^ showed that arecoline, a mAChR agonist, induced neuronal death by apoptosis at concentrations from 50–200 μM. The generation of reactive oxygen species, the decrease in antioxidant defenses and the activation of caspase-3 were some of the mechanisms studied. Besides the chemical similarity among AEME and arecoline, with similar pK_i_ values in binding studies on cloned M_1_-M_5_ mAChRs^25^, it seems that these substances share the same neuronal death pathways, culminating in the activation of caspase-3^16^,^24^. This executioner enzyme can mediate the catabolic process, characterizing the end-stage of apoptosis^26^. The current manuscript corroborates with this finding, suggesting that one of the mechanisms involved in DNA fragmentation observed 24 hours of exposure to 10^−3^ M AEME could be explained by the previous activation of caspase-3. Whether, the DNA fragmentation might be a consequence of this intracellular cascade activation remains to be explored.

AEME-induced neurotoxicity could be triggered by an intracellular calcium increase, in a concentration-dependent manner, observed at concentrations starting at 10^−4^ M (100 μM). The endoplasmic reticulum contains calcium release channels which can be activated by IP_3_ through IP_3_ receptors^27^. Thus, the increase in free cytosolic calcium observed at AEME concentrations starting at 10^−7^ M (0.1 μM) and reaching a maximum at 10^−5^ M (10 μM) may be triggered by the increases in inositol phosphate accumulation, in particular IP_3_. This outcome may be explained by the partial agonist effect at M_1_ and M_3_ mAChRs as observed in the *in vitro* CHO cells data for AEME. It is important to emphasize that the AEME-induced neurotoxicity investigated by Garcia *et al.*^16^ occurred after a long-term exposure, i.e., different concentrations of AEME for 12 and 24 hours.

Few studies have evaluated the effects of AEME at mAChRs^13^,^14^,^15^. Our group was the first one to correlate the neurotoxic effects of AEME with the activation of mAChRs, as the nonspecific mAChR antagonist atropine was able to prevent AEME-induced neurotoxicity^16^. Although M_4_ and M_5_ mAChRs subtypes are less expressed in the hippocampus^19^,^20^ and AEME showed an antagonist effect when interacting with these subtypes, we believe that these effects are not likely to explain AEME-mediated neurotoxicity because of the preventive atropine effect^16^. However, we cannot discard their importance. M_2_ mAChRs subtypes are found in this brain region in cholinergic synaptic terminals controlling acetylcholine and other neurotransmitter release, e.g., glutamate, which could promote excitotoxicity at high concentrations^28^. Also, some substances with an antagonist effect at the M_4_ mAChR subtype, when injected in the dorsal hippocampus of rats, induce retrograde amnesia, disrupting memory consolidation^29^.

Several substances, e.g., the muscarinic toxins (MTs), a group of small proteins isolated from the venom of some snakes, have a high selectivity and affinity for individual mAChRs subtypes with competitive antagonist, allosteric modulator, and potential agonists effects^30^. MT2 toxin, for example, activates M_1_, M_3_ and M_5_ mAChRs, leading to a significant increase in intracellular calcium^23^. According to Bashkatova *et al.*^31^, the stimulation of M_1_ mAChRs by the injection of M_1_ agonist McN-A-343 increased nitric oxide and lipid peroxidation in the striatal tissues, the same effect observed for the psychostimulant amphetamine. Several cells, including neurons, present the constitutive form of nitric oxide synthase, which is a calcium/calmodulin-dependent enzyme rapidly activated in response to intracellular calcium increase, leading to nitric oxide production^32^,^33^. Depending on the pathophysiological conditions, it could be overproduced, resulting in cellular toxicity and death by oxidative stress and lipid peroxidation^34^. Interestingly, MT7, a potent non-competitive antagonist toxin at the M_1_ mAChR subtype, with no antagonist activity at the M_3_ or M_5_ mAChRs subtypes^23^, was able to prevent amphetamine-induced nitric oxide generation and the lipid peroxidation process. The authors attributed that the activation of M_1_ mAChRs might play a critical role in the neurotoxic process induced by amphetamine^31^. Our previous study showing the preventive effect of mAChRs antagonist atropine in the AEME-induced neurotoxicity^16^ corroborate with these findings. Moreover, we demonstrated that M_1_ selective antagonist (pirenzepine) and the M_3_ selective antagonist (p-F-HHSiD), were able to prevent or reduce AEME-induced neurotoxicity. In addition, the PLC inhibitor U73122 was also able to prevent AEME toxicity indicating that these effects are mediated by PLC activation. Taking together, these results indicate that the neurotoxic effects of AEME may involve the activation of both M_1_ and M_3_ mAChRs.

To the best of our knowledge, this is the first study demonstrating AEME mechanism of action at the mAChRs. Its partial agonist effect at M_1_ and M_3_ mAChRs may be the cause of AEME neurotoxicity, once the selective antagonists were able to prevent it. The IP_3_ accumulation and the increase in free cytosolic calcium, as well as oxidative stress, could result in mitochondrial dysfunction, affecting the electron transfer chain, leading to ATP depletion and neuronal death by caspase activation, which causes DNA fragmentation^35^.

This study corroborates with our previous study, reinforcing the idea that AEME is more than a crack cocaine biomarker; it may play a crucial role in several CNS disorders, including cognitive deficits of crack cocaine users^36^, as they are exposed to a mixture of cocaine, AEME and others (e.g. solvents) that could enhance the risk of a neurotoxic effect.

## Material and Methods

### Anhydroecgonine methyl ester (AEME)

Cocaine was gently donated by the Criminal Institute of São Paulo to the Laboratory of Toxicological Analyses (School of Pharmaceutical Sciences, University of São Paulo) for research purposes. Briefly, cocaine was purified (95%) and converted into its salt form, cocaine hydrochloride, by bubbling hydrochloric acid into mixture of purified cocaine dissolved in diethyl ether. Then, AEME was synthesized, using cocaine hydrochloride as start material and purified as previously described by our research group^16^. The AEME product (purity >98%) was confirmed by proton nuclear magnetic resonance (^1^H-NMR) and electrospray ionization-mass spectrometry (ESI-MS). For the muscarinic CHO cells studies, AEME was purchased from Lipomed^®^ (purity >98%).

### Animals

Pregnant *Wistar* rats, weighing 230–250 g, were obtained from Butantan Institute, São Paulo, Brazil. They were housed in plastic cages and maintained in a room with constant temperature (22 ± 1 °C) on a 12:12 hours light/dark cycle (lights on at 7:00 a.m.). Food and water were provided *ad libitum*. This study was performed according to NIH guidelines and approved by Animal Use Ethic Committee of Butantan Institute (protocol number 633/09) and Ethic Committee for Research Project Analysis of School of Medicine at University of São Paulo (protocol number 0841/09).

### Experiments with rat primary hippocampal cell culture

#### Hippocampal cell culture and immunohistochemical characterization

Hippocampal neurons were dissociated from hippocampi of E18-E19 Wistar rat embryos, as described previously^37^,^38^,^39^,^40^. Pregnant rats were anesthetized with sodium pentobarbitone 55 mg/kg and the fetuses were rapidly decapitated to remove their hippocampi. The tissue was placed into a Petri dish containing 100 U/mL penicillin and 100 μg/mL streptomycin (Gibco) in a cooled Neurobasal medium (Gibco). Hippocampi were washed with Hank’s Balanced Salt Solution (HBSS) and submitted to a mechanical fragmentation using appropriate scissors. Hippocampi fragments were then transferred to a 0.25% trypsin in Earl’s Balanced Salt Solution (EBSS) solution pH 7.2–7.4 and were incubated for 10 minutes at 37 °C. After the incubation period, cells were washed with an EBSS solution containing 277.5 U/mL DNAse (Sigma) and 10% fetal bovine serum (FBS) (Gibco) and centrifuged at 300 *g* (Eppendorf 5804R) for 2 minutes at 20 °C. Neurons were isolated by mechanical dissociation in an EBSS solution (with DNAse and fetal bovine serum) using Pasteur pipettes with different diameters sizes and centrifuged for 5 minutes (300 *g*). The tissue was then resuspended in Neurobasal medium (Gibco) supplemented with 0.5 mM L-glutamine, 25 μM L-glutamic acid, 100 U/mL penicillin, 100 μg/mL streptomycin and 2% B27 supplement (Gibco) to reduce glial cell proliferation^40^,^41^. The cells were seeded onto 0.01% poli-L-lysine-coated multiwell culture plate and maintained at 37 °C in a humidified atmosphere of 5% CO_2_, for 7–8 days, the time required for maturation of hippocampal neurons, forming a network of functional synaptic contacts^42^. On the second day, half of the old medium was replaced by the same volume of a fresh medium with the same composition. On the seventh day, cells were incubated with AEME in several concentrations for different time periods, depending on the experiment. Hippocampal neurons were plated on poli-L-lysine-coated 24-well culture plate at a density of 2 × 10^5^ cells/cm^2^. The culture cells were immunohistochemically characterized with MAP2 (neuronal marker) and GFAP (astrocytic marker) showing a predominance of 92% of neurons and 8% of astrocytes^16^,^43^.

#### Effect of AEME on total [^3^H]inositol phosphates accumulation

Hippocampal cells (1 × 10^6^ cells/well) were allowed to equilibrate for 10 minutes with a nutrient solution of the following composition (mM): NaCl 157.00; KCl 5.60; CaCl_2_ 0.27; MgCl_2_ 3.00; NaHCO_3_ 1.80; glucose 5.50 (pH 7.0–7.2) at 37 °C under constant shaking. Cells were incubated with 5 μCi of myo[^3^H]inositol (specific activity 18.0 Ci/mmol) for 80 minutes, and lithium chloride (10 mM) for additional 30 minutes. Afterwards, the cells were incubated in the absence (basal level) and in the presence of carbachol (positive control) and AEME (10^−8^ to 10^−3 ^M) for 10 minutes. Cells were removed from the plate using a 0.1 M NaOH solution and washed three times with nutrient solution, transferred to 2 mL of methanol:chloroform (2:1 v-v) at 4 °C and homogenized with a Ultra-Turrax T25 homogenizer at 9500 rpm. Chloroform (0.62 mL) and H_2_O (0.93 mL) were added to the homogenate, and the solution was centrifuged for 5 minutes at 1,000× *g* to separate aqueous and organic phases^44^. Total [^3^H]inositol phosphate was measured as previously described by Ascoli *et al.*^45^ with slight modification. The aqueous layer was neutralized with 0.1 M HCl and then mixed with 1 mL anion-exchange resin (Dowex AG-X8, formate form, 200–400 mesh), allowed to equilibrate for 30 minutes at room temperature, under agitation, and centrifugated at 1,000× *g*, for 5 minutes at 4 °C. The resin was then washed sequentially, with 10 mM myo-inositol (2 mL) and 5 mM sodium tetraborate/60 mM sodium formate (2 mL). The resin was incubated for 30 minutes at room temperature with 2 mL of 0.1 M formic acid/1 M ammonium formate). The total [^3^H]inositol phosphate was eluted and placed in scintillation vials containing OptiPhase HiSafe 3. The amount of radioactivity was determined in scintillation β-counter (LS 6500 IC, Beckman). Total [^3^H]inositol phosphate was expressed as percentage above basal level.

#### Effect of AEME on intracellular calcium release

Hippocampal cells were removed from the plates and previously incubated with Fluo4-AM dye (Invitrogen) dissolved in Neurobasal medium (Gibco®) for one hour^46^. The cells were centrifuged (300 *g* for 5 minutes at 20 °C), washed with phosphate buffer solution (PBS) without calcium, and plated in microscopy confocal (Zeiss LSM 510, Meta) microplates pretreated with poli-L-lysine. The fluorescence was measured for 90 seconds in the absence and/or in the presence of 10^−5^, 10^−4^ and 10^−3^ M AEME. After this period, 250 mM KCl was added in order to promote neuronal death and, thus, verify its viability. All measurements were performed with a PBS free-calcium solution. Digitalized images were analyzed through the software *Origin 5*.

#### Effects of AEME on DNA fragmentation

DNA fragmentation was assessed using propidium iodide following the method described by Lima *et al.*^47^. Hippocampal cells were incubated with 10^−4^ or 10^−3^ M AEME for 12 and 24 hours. Briefly, exposed cells were removed from the culture plate and centrifuged (500 *g* for 5 minutes) and lysed with a fragmentation buffer (100 mg/mL propidium iodide, 0.1% sodium citrate, and 0.1% Triton-X) for 2 hours at room temperature in the dark. DNA fragmentation was analyzed in a flow cytometry (FACSCalibur, Becton Dickinson, CA) using the FL2-A channel (λ_excitation_ = 585 nm and λ_emission_ = 642 nm) and 10 million cells were evaluated per experiment.

### Experiments with CHO cells

#### CHO cell culture

Chinese hamster ovary (CHO) cells individually and stably expressing individual rat mAChRs (rM_1_ to rM_5_ mAChRs) were purchased from the American Type Culture Collection (ATCC) and cultured according to ATCC recommendations. To generate stable rM_2_ and rM_4_ cell lines for use in calcium mobilization assays, rM_2_ and rM_4_-expressing cells were stably transfected with a chimeric G protein (G_qi5_) using Lipofectamine 2000 (Invitrogen). rM_2_ and rM_4_ cells were grown in Ham’s F-12 medium containing 10% heat-inactivated fetal bovine serum (FBS), 2 mM GlutaMAX (Gibco®), 20 mM HEPES, 500 μg/mL G418 sulfate, 200 μg/mL hygromycin B and an antibiotic-antimycotic solution (Gibco®). rM_1_, rM_3_ and rM_5_ mAChR-CHO cells were grown in the same medium without hygromycin B^48^. CHO cells were cultured in a 150 mm-diameter cell culture-treated dishes (Corning®) at 37 °C and 5% CO_2_ until complete confluence.

#### Membrane preparation and equilibrium radioligand binding assays

Membranes were prepared from CHO cells stably expressing individual rat mAChRs (rM_1_ to rM_5_). Briefly, media was removed, cells were washed with PBS and harvested with PBS into a centrifuge bottle. Cells were spun at 2,000 *g* for 5 minutes at 4 °C. After harvesting, the supernatant was gently discarded and cold membrane preparation buffer [Hanks’ balanced salt solution (HBSS; Invitrogen), 4.17 mM sodium bicarbonate, 20 mM HEPES and 0.5 mM EDTA] was added (10 mL/dish). Cells were then homogenized for 5–10 seconds with a rotary homogenizer (Polytron®) and centrifuged at 20,000*g* for 20 minutes at 4 °C. The homogenization/centrifugation cycle was repeated twice. After the last centrifugation, the supernatant was gently discarded and the pellet was resuspended in cold membrane preparation buffer (up to 2 mL). All binding reactions were performed in a total volume of 1 mL containing 25 μg of membrane protein. Non-specific binding was determined in the presence of 1 μM atropine. l-[N-methyl-^3^H]scopolamine ([^3^H]NMS; GE Healthcare) saturation binding was performed to calculate both the index of the receptor density (B_max_) and the NMS dissociation constant (NMS K_d_) values for each subtype of mAChR. The competition binding reactions were carried out in 96-well deep-well plates with membrane protein in the presence of appropriate concentrations of AEME or vehicle, and 0.1 nM [^3^H]NMS for 3 hours at room temperature. The equilibrium binding was terminated by rapid filtration using a 96-well harvester (Brandel®). Filters were washed three times with ice-cold harvesting buffer and were dried overnight. Radioactivity was counted using a TopCount NXT (PerkinElmer®) and counts were normalized to the maximal specific binding in the presence of vehicle^48^,^49^,^50^. Saturation and competition binding data were analyzed using a weighted nonlinear least-squares interactive curve-fitting program GraphPad Prism (GraphPad Prism Software version 5.0). A mathematical model for one or two binding sites was applied. The equilibrium dissociation constant (K_D_) and the binding capacity (B_max_) were determined^51^. The inhibition constant (K_i_) was determined from competition curves using the Cheng and Prusoff equation^52^.

#### Calcium mobilization assay

CHO cells expressing rM_1_, rM_3_ and rM_5_ were plated at 4 × 10^4^ cells per well, whereas rM_2_ and rM_4_ were plated at 6 × 10^4^ cells per well, in standard growth media (as described previously under “cell culture”) in 96-well plates 24 hours before assay and were incubated overnight at 37 °C in 5% CO_2_. On the day of the assay, media was removed, cells were washed with calcium assay buffer [Hanks’ Balanced Salt Solution (HBSS; Invitrogen), 20 mM HEPES, 4.17 mM sodium bicarbonate, 2.5 mM probenecid (Sigma), pH 7.4] and maintained in calcium assay buffer containing 2.3 μM Fluo4-AM dye (Invitrogen). Cells were incubated for 45 minutes (37 °C, 5% CO_2_) for dye loading. Fluo4-AM dye was removed, cells were washed and replaced with 40 μL of calcium assay buffer. AEME concentration-response curves were determined in single-add experiments. For calcium fluorescence measurements of AEME potency (“double-add” calcium assay), 100 μM AEME was added 20 seconds after the beginning of data collection and increasing concentrations of acetylcholine were added 100 seconds later via Flexstation II (Molecular Devices). Fluorescence measurement continued for a total of 200 seconds of acquisition time using an excitation wavelength of 488 nm and an emission wavelength of 525 nm. For Schild analyses, fixed concentrations of AEME (10^−6^ to 10^−3.5^ M) were added before the acetylcholine concentration-response curve in the same way described previously for the “double-add” calcium assay. All compounds were dissolved in dimethyl sulfoxide (DMSO) and then in calcium assay buffer to obtain a final DMSO concentration of 0.3%. All of the peaks of the calcium response were normalized to baseline and then as a percentage of the maximum acetylcholine response. These values were fit using GraphPad Prism version 5.0 to determine EC_50_ values and Schild slope and pA2 in the Schild analyses^48^,^53^,^54^.

### Effect of M_1_ and M_3_ selective mAChR antagonists and the PLC inhibitor on AEME-induced neurotoxicity

Neuronal viability was evaluated in the hippocampal cell culture using the 3-(4,5-dimethylthiazol-2-yl)-2,5-diphenyltetrazolium bromide (MTT; Sigma) reduction assay, with some modification^16^. Briefly, after 24 hours of incubation with 10^−3 ^M AEME in the absence and in the presence of 10 nM pirenzepine hydrochloride (M_1_ selective antagonist; Sigma), 10 nM p-F-HHSiD (M_3_ selective antagonist; Sigma) and the specific inhibitor of different isoforms of PLC U73122 (10 nM)^55^ (Sigma), all the medium was removed, and 100 μL of MTT solution, containing 5 mg/mL MTT in PBS and neurobasal medium without phenol red (1:9, v/v) were added. After 3 hours of incubation with MTT at 37 °C in a humidified atmosphere of 5% CO_2_, the MTT solution was removed and 200 μL of dimethyl sulfoxide was added to each well. After 30 minutes of shaking, the absorbance was measured at 570 nm in a multiwell plate reader (BioTek Synergy H1 Hybrid Reader). Potassium chloride (250 mM) was used as a positive control of neuronal death. Muscarinic antagonists, as well as the iPLC, were added 30 minutes prior to incubation with AEME. The antagonist concentration used was near the pK_i_^25^,^56^. A control containing 0.002% DMSO was used, since the iPLC stock solution was previously dissolved in this solvent. The assay was performed in quadruplicate, and the results were expressed as a percentage of the control value^16^.

### Statistical analyses

Data were analyzed by one-way analysis of variance (ANOVA) followed by Newman-Keuls multiple comparison *post-hoc* test. Acetylcholine concentration-response curve in the presence of 100 μM AEME was analyzed by Student’s *t*-test. *P* < 0.05 was considered statistically significant. All data were plotted and analyzed by GraphPad Prism software version 5.0. Data were reported as *mean*  ±  *SEM*.

## Additional Information

**How to cite this article**: Tamborelli Garcia, R. C. *et al.* M_1_ and M_3_ muscarinic receptors may play a role in the neurotoxicity of anhydroecgonine methyl ester, a cocaine pyrolysis product. *Sci. Rep.* **5**, 17555; doi: 10.1038/srep17555 (2015).

## Supplementary Material

Supplementary Information

## Figures and Tables

**Table 1:** Saturation binding experiment of [^3^H]NMS in separate CHO cells expressing one of the five subtypes of mAChRs.

*mAChR subtype*	B_max_ (fmol/mg of protein)	K_d_ (×10^-3^ μM)
Rat M_1_	1087 ± 165	0.085 ± 0.004
Rat M_2_	1470 ± 053	0.159 ± 0.022
Rat M_3_	1132 ± 033	0.063 ± 0.008
Rat M_4_	1835 ± 127	0.038 ± 0.004
Rat M_5_	1564 ± 027	0.224 ± 0.016

B_max_ and K_d_ values were determined for radioligand competition binding using *GraphPad Prism 5* (data represent the mean of three independent experiments performed in triplicate). Data presented as mean ± SEM.

**Table 2:** The EC_50_ for acetylcholine concentration-response curve in the absence and in the presence of 100 μM AEME (data represent the mean of three independent experiments, each one performed in duplicate).

*mAChR subtype*	EC_50_ in nM (mean ± SEM)	
	ACh–DMSO control	ACh concentration-response curve + 100 μM AEME
**Rat M**_**1**_	11.5 ± 0.6	63.1 ± 2.5^***^
**Rat M**_**2**_	123.0 ± 9.8	263.0 ± 21.0^*^
**Rat M**_**3**_	6.5 ± 0.4	34.7 ± 2.8^**^
**Rat M**_**4**_	166.0 ± 14.9	524.8 ± 47.2^*^
**Rat M**_**5**_	4.3 ± 0.4	29.5 ± 3.0^**^

^*^p < 0.05, ^**^p < 0.01 and ^***^p < 0.001, compared with ACh concentration-response curve alone for each mAChR subtype. mAChR: muscarinic acetylcholine receptor, DMSO: dimethyl sulfoxide, ACh: acetylcholine.

Results are presented as mean ± SEM in nM.
