# Supplementary material for: M1 and M3 muscarinic receptors may play a role in the neurotoxicity of anhydroecgonine methyl ester, a cocaine pyrolysis product
Source: Sci Rep. 2015 Dec 2;5:17555. doi: 10.1038/srep17555 (PMC4667193; doi:10.1038/srep17555)
Supplement: Supplementary Information [file srep17555-s1.pdf]

M_1 and M_3 muscarinic receptors may play a role in the neurotoxicity of anhydroecgonine methyl ester, a cocaine pyrolysis product

Raphael Caio Tamborelli Garcia[1,2,6,7†], Livia Mendonça Munhoz Dati[1†], Larissa Helena Torres[1], Mariana Aguilera Alencar da Silva[1], Mariana Sayuri Berto Udo[1], Fernando Maurício Francis Abdalla[3], José Luiz da Costa[4], Renata Gorjão[5], Solange Castro Afeche[3], Mauricio Yonamine[1], Colleen M. Niswender[6,7], P. Jeffrey Conn[6,7], Rosana Camarini[8], Maria Regina Lopes Sandoval[3], Tania Marcourakis[1,*]

[1]Department of Clinical and Toxicological Analysis, School of Pharmaceutical Sciences, University of São Paulo, Av. Prof. Lineu Prestes, 580, Bl. 13B, 05508-000, São Paulo/SP, Brazil.
[2]Institute of Environmental, Chemical and Pharmaceutical Sciences, Federal University of São Paulo, Rua São Nicolau, 210, 1° andar, 09913-030, Diadema/SP, Brazil.
[3]Laboratory of Pharmacology, Butantan Institute, Av. Vital Brasil, 1500, 05503-900, São Paulo/SP, Brazil.
[4]Criminalistic Institute of São Paulo, Rua Moncorvo Filho, 410, 05507-060, São Paulo/SP, Brazil.
[5]Institute of Physical Activity Sciences and Sports, Post-Graduate Program in Human Movement Sciences, Cruzeiro do Sul University, São Paulo, Brazil.
[6]Department of Pharmacology, Vanderbilt University Medical Center.
[7]Vanderbilt Center for Neuroscience Drug Discovery, Vanderbilt University Medical Center, 2201 West End Avenue, 1205 Light Hall, 37232-0697, Nashville/TN, USA.
[8]Department of Pharmacology, Institute of Biomedical Sciences, University of São Paulo, Av. Prof. Lineu Prestes, 1524, Prédio 1, 05508-900, São Paulo/SP, Brazil.
Contact email: tmarcour@usp.br
[*]Corresponding author.
[†]These authors contributed equally to this work.

Address for correspondence
Faculdade de Ciências Farmacêuticas da Universidade de São Paulo, Av. Prof. Lineu Prestes, 580, Bl. 13B, CEP 05508-000, São Paulo/SP, Brazil.
Tel/Fax: +55-11-3091-1504
Email: tmarcour@usp.br (T.Marcourakis)

Saturation binding of [^{3}H]NMS for each mAChR subtype expressed in CHO cells.

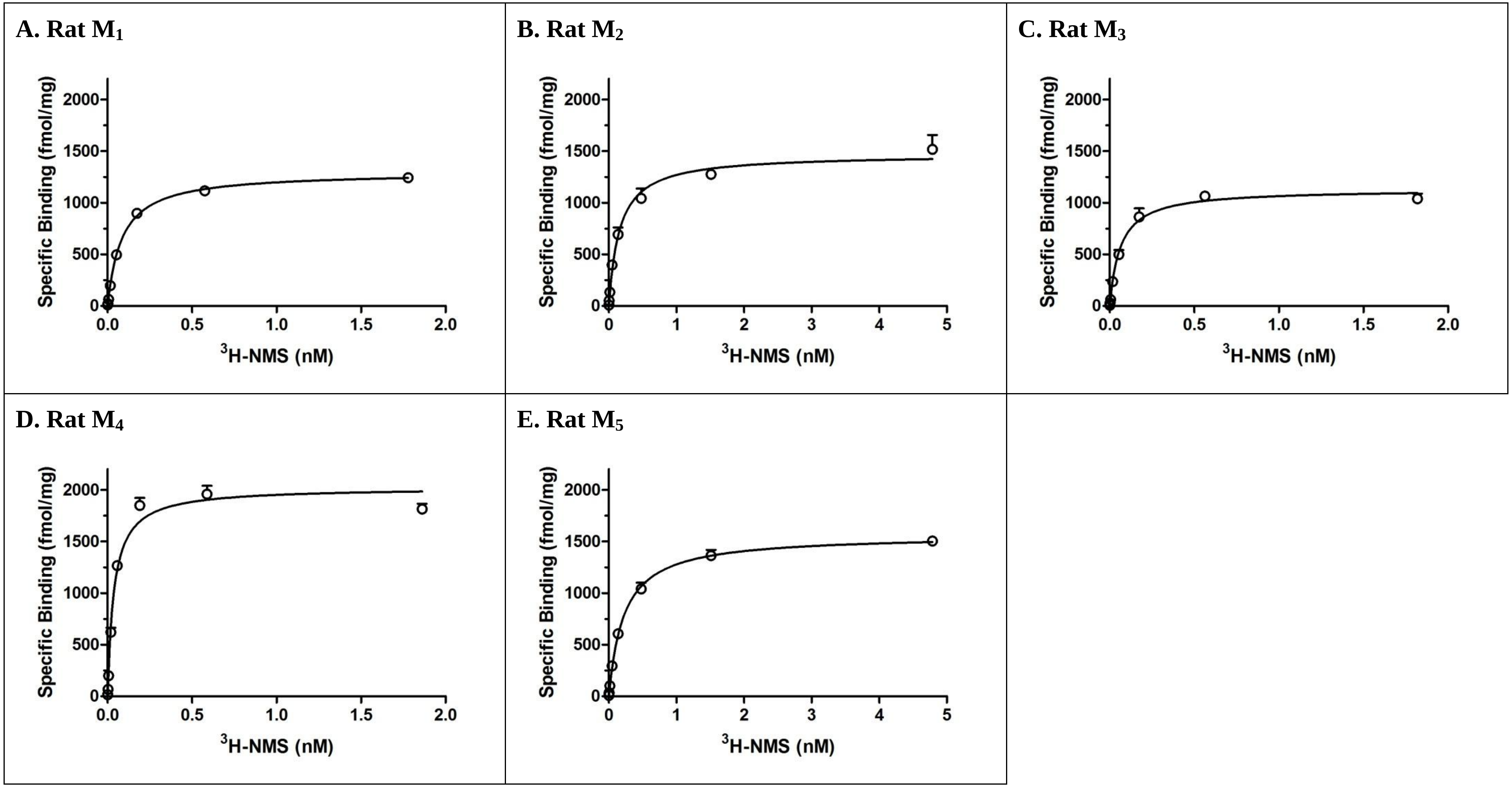

Supplementary Figure S1. Saturation binding curves (data represent the mean of three independent experiment performed in triplicate) of [^{3}H]NMS for each mAChR subtype expressed in CHO-K1 cells. B_{max} and K_d values were determined for radioligand competition binding using *GraphPad Prism 5*. **A.** Rat M_1 (B_{max}= 1087±165 fmol/mg of protein; K_d=0.085 nM); **B.** rat M_2 (B_{max}= 1470±53 fmol/mg of protein; K_d=0.159 nM); **C.** rat M_3 (B_{max}= 1132±33 fmol/mg of protein; K_d=0.063 nM); **D.** rat M_4 (B_{max}= 1835±127 fmol/mg of protein; K_d=0.038 nM) and **E.** rat M_5 (B_{max}= 1564±27 fmol/mg of protein; K_d=0.224 nM. Data presented as mean±SEM.
